# Supplementary material for: Sea surface temperature modulates El Niño and La Niña driven leptospirosis patterns: Evidence from causal machine learning in Colombia
Source: PLOS Glob Public Health. 2025 Nov 6;5(11):e0005238. doi: 10.1371/journal.pgph.0005238 (PMC12591457; doi:10.1371/journal.pgph.0005238)
Supplement: Attachment — Submitted filename: Response_to_Reviewers.docx [file pgph.0005238.s002.docx]

Journal Requirements:

Figure 3: please (a) provide a direct link to the base layer of the map (i.e., the country or region border shape) and ensure this is also included in the figure legend; and (b) provide a link to the terms of use / license information for the base layer image or shapefile. We cannot publish proprietary or copyrighted maps (e.g. Google Maps, Mapquest) and the terms of use for your map base layer must be compatible with our CC-BY 4.0 license.

R: We appreciate your comment. However, Figure 3 does not contain any copyrighted material, and we have added the following text to the legend: “The map was created using QGIS software, with the basemap shapefile sourced from the Colombian National Geostatistical Framework, an openly available resource (https://www.dane.gov.co/files/geoportal-provisional). The terms of use for the base shapefile are compatible with CC-BY 4.0 (https://geoportal.dane.gov.co/acerca-del-geoportal/licencia-y-condiciones-de-uso/#gsc.tab=0)”

Reviewer #1:

The article “Sea Surface Temperature Modulates El Niño and La Niña Driven Leptospirosis Patterns: Evidence from Causal Machine Learning in Colombia” examines the relationship between El Niño and La Niña episodes and leptospirosis cases in Colombia at the municipal level from 2007 to 2023 using a Casual Machine Learning approach.

The data and methodology used are appropriate and correctly applied, and the originality of the work arrives from the inverse of what is already accepted in the scientific community. In general, leptospirosis epidemics are driven by humid events, such as those caused by La Niña periods in the study region, and the opposite occurs during drier periods, but this work shows the contrary. Also, the novelty of the work is that it used real case records over an extended period in a region where there was no published evidence of the relationship between climate and this disease.

In order to be accepted, I consider that the quality of figures and tables should be improved. All of them present low definition and contrast of colors.

R: We appreciate your comment, and we have improved the contrast of the colors of the figures. However, note that the figures have a resolution of 300 ppi, which meets the journal's requirements.

Specifically, Figure 2 could show higher detail of the years of data at the x-axis, as well as remark the years with more important outbreaks. In the caption, clarify if the data correspond to the whole country or some specific municipalities.

R: We appreciate your comment. We have improved the details of the years on the x-axis and highlighted the months with the highest number of cases. Additionally, we clarified in the caption that the data correspond to the total number of cases in the entire county.

Additionally, in Figure 3, please indicate the names of the municipalities with the highest number of cases, to correspond with the text of the article.

R: We have changed Figure 3 to indicate the names of the municipalities with the highest number of cases (Fig. 3a).

On the other hand, Table 2 would be more legible if it appeared on a single page, perhaps with a smaller font size. Please consider redesigning it.

R: We appreciate your comment, and we have changed the font size of Table 2.

You must say which works of literature you are referring to on line 456.

R: Dear Reviewer, on line 456, we are not referring to specific previous works because, in this sentence, we clarify that our approach to achieving consensus across the four climate agencies (NOAA, MOAG, TCC, and IDEAM) for identifying ENSO episodes is intended to satisfy the consistency assumption of causal inference. However, due to the absence of previous studies with a similar approach, earlier works did not address the consistency assumption, and thus cannot be cited.

For future work, it is recommended to analyze the impact of ENSO phenomena at local or regional scale with similar approaches, to take into account regional heterogeneity in climate-health associations.

R: We appreciate the comment and have included a new phrase (lines 690-692) to suggest this point in future works.

Reviewer #2:

This manuscript investigates the causal effects of El Niño and La Niña climatic events on leptospirosis incidence in Colombia using an ecological longitudinal design and a causal machine learning framework with doubly robust estimation and overlap weighting. The study spans data from 2007 to 2023, integrating epidemiological surveillance records, satellite-derived climate data, and socioeconomic indicators to estimate both Average Treatment Effects (ATE) and Conditional Average Treatment Effects (CATE).

- Critical methodological components are not well explained or justified, particularly regarding treatment definition, model assumptions, and how excess cases are calculated.

R: We appreciate your comment. We have revised the Methods section to clarify key aspects, including the definition of treatment, model assumptions, and the calculation of excess cases. Please see the details in the answers below.

- Presentation of figures and tables is inconsistent or unclear, with some results hard to interpret or misaligned with the stated goals (e.g., monthly trends shown as annual aggregates).

R: We appreciate your comment. We have improved Figure 2 and Table 2, as mentioned in the specific comments (see answers below).

- Key robustness and sensitivity tests are underdeveloped or raise questions about statistical validity, particularly Table 2 which appears internally inconsistent.

R: We appreciate your comment a lot, because it allows us to detect an error in the code, related to the refutation tests shown in Table 2. Additionally, we have improved the Methods section to clarify the robustness tests implemented and have included new paragraphs to facilitate the interpretation of Table 2 (see the details of our responses in the specific comments).

- The causal claims, especially the interpretation of CATE and refutation tests, are not sufficiently supported or explained, leading to ambiguity around the main findings.

R: We appreciate your comment. We have improved the Methods and Results sections to clarify the causal claims related to the CATE and the refutation tests implemented. You can find the details of our responses in the specific comments.

Major Comments:

- Figure 2-is labeled as "Monthly time series of leptospirosis cases", yet the actual visualization appears to aggregate data annually. If monthly variation is central to your modeling (e.g., to match ENSO phases), the data resolution in the figure should reflect that. Otherwise, the figure is misleading and inconsistent with the analysis frame.

R: We appreciate your comment. We have improved the details on the x-axis (Time) and highlighted the months with the highest number of cases, as suggested by the other reviewer.

- It remains unclear how ENSO exposure is temporally aligned with disease cases. Are cases lagged behind SST anomalies? If so, what lag window was used? Causal claims hinge on this temporal structure and must be justified more rigorously.

R: We appreciate your comment. Please note that the cases are not lagged behind SST anomalies because we used the consensus of four climate agencies (NOAA, TCC, MOAG, and IDEAM) as the criterion to define episodes of El Niño, La Niña, and Neutral. Additionally, we have modified Section 2.2, Outcome and Exposure Variables (lines 224-242), to clarify how ENSO exposure is temporally aligned with the outcome variable.

- The paper references use of doubly robust estimation and overlap weighting but provides insufficient detail on the implementation. Which model was used for the outcome regression? What was the propensity model? Were these trained separately or in a joint framework?

R: We appreciate this important point. Note that to address potential violations of the positivity assumption, we estimate first the propensity score and the overlap weights. However, due to computational restrictions, we implemented a logistic regression with Ridge regularization for this estimation. Then, to estimate the effect, we used a double robust estimation approach, employing a random forest for the outcome regression and propensity score modeling, in a joint framework. We have modified Section 2.5, Machine Learning Implementation (lines 336-393), to clarify this point.

- More importantly, how is “excess leptospirosis” defined for the treatment effect estimation? The phrase is repeatedly used, but the operational definition (e.g., above mean? above baseline trend? z-score threshold?) is not formally described. This is critical for reproducibility and interpretability.

R: We appreciate the comment. Please note that now lines 136-171 explain the process of obtaining the operational definition of excess cases. In summary, to identify excess cases of leptospirosis, we first estimated the Standardized Incidence Ratio (SIR) with the R package epitools. The SIR compares the observed number of disease cases in a target population to the expected cases based on a reference population, typically at the national level. An SIR value greater than 1 indicates a higher-than-expected occurrence of the disease in the municipality, while values below 1 suggest a lower incidence compared to the reference population. In this context, to determine if a municipality has excess leptospirosis cases in a specific month, we binarized the SIR, assigning a value of 1 when the SIR was greater than 1 (i.e., excess cases TRUE) and 0 otherwise (i.e., excess cases FALSE).

- This table is particularly confusing. For example, some rows show "estimated effect = -0.010" with no explanation of units or interpretation. Why do some entries show 0.000 exactly? Is this due to rounding, or lack of effect? Also, the statistical significance labeling (p-values in bold) is introduced without a clear link to model diagnostics. What are these p-values testing—significance of the ATE or a test for hidden confounding (e.g., placebo/refutation analysis)? Please clarify.

R: We appreciate your comment. As we mentioned above, your feedback has allowed us to identify an error in the code: we were not including the overlap weights in the refutation tests. We have resolved this issue. Note that we repeated each refutation 50 times. In all tests, the p-value assesses whether the new effect estimate under the perturbation induced by the refutation test is statistically different from the original estimate (or from zero, in the case of the placebo and random outcomes). Additionally, we have modified Section 2.6, Robustness tests in Methods (lines 394-449), and the results (lines 536-563) to clarify the interpretation of Table 2.

- Additionally, the meaning of “latent bias” is vague. Are you conducting a sensitivity analysis (e.g., Rosenbaum bounds)? Or are these refutation tests based on synthetic controls or negative outcome models? The term “refutation” needs to be grounded in methodology.

R: We appreciate your comment. We have changed the term “latent bias” to “remaining bias” to avoid confusion in the readers. Note also that our refutation tests are based on negative synthetic controls. Additionally, we have modified Section 2.6, Robustness tests in Methods (lines 394-449), to clarify the meaning of “remaining bias” in the context of refutation tests and to highlight its difference from the sensitivity analysis of Rosenbaum bounds.

- The paper claims that "as sea surface temperatures rise, the impact of ENSO diminishes." This is an interesting but highly speculative claim unless directly supported by CATE visualization or conditional models that include interaction terms. If you used sea surface temperature as a moderator, please show actual conditional effect plots. Without this, the claim risks overstating the evidence.

R: Please note that we are not using a regression approach to estimate the effect, nor an interaction term to estimate the change in the effect produced by SST. Instead, we have implemented a causal machine learning framework (a non-parametric approach); for this reason, we estimate the change in the effect produced by SST12 as a conditional effect. Additionally, note that lines 515-527 and Figure 5 explain the results of the CATE conditioned on SST12 (i.e., in front of the Colombian Pacific coast) for the three scenarios studied. This means that our claim about the relationship between the effect of ENSO episodes and sea surface temperature is limited to SST12. In addition, please note that SST12 was included in the model as a potential confounder, as shown in Figure 1 and Table 1, and as the effect modifier to estimate the CATE. Furthermore, we included a new paragraph (lines 377-389) to provide a detailed explanation of the CATE estimation procedure.

- Also, if SST is itself driving the definition of El Niño/La Niña, how is it being separately used as a conditioning variable in CATE? This may introduce post-treatment bias if not handled carefully.

R: We appreciate your comment. Please note that we are conditioning only on SST12. Additionally, we have modified Section 2.1.2, Environmental Data (lines 172-204), to clarify the definition used by each climate agency (NOAA, TCC, MOAG, and IDEAM) to identify an El Niño, La Niña, or Neutral episode. In these lines, you can see that SST12 is not a criterion used by any climate agency.

- How are El Niño and La Niña episodes operationalized? Are you using NOAA’s ONI thresholds? Were partial years discarded? How are ambiguous or transitional periods handled? This is fundamental to the treatment/control assignment, and currently left unspecified.

R: As mentioned above, we have included the definition used by each climate agency to identify an El Niño, La Niña, or Neutral episode. Additionally, in lines (172-195), we explain that to fulfill the assumption of consistency, we used the consensus among all four climate agencies (NOAA, TCC, MOAG, and IDEAM) to operationalize El Niño, La Niña, and Neutral episodes in our study. Furthermore, we show a couple of examples of how definitions of ENSO episodes, made without the consensus of the four climate agencies, lead to violations of the consistency assumption of causal inference (lines 325-335). Note that the Supplementary_S1 (mentioned in line 193) shows, for each month between 2007 and 2023, whether the month was classified as part of an El Niño, La Niña, or Neutral episode, for each climate agency, along with the corresponding consensus.

Additional Concerns:

- Figure labeling and terminology: Please ensure consistency in terminology—"ENSO events" vs. "ENSO phases", "excess cases" vs. "probability of excess cases", etc.

R: We appreciate the comment. We have ensured consistency in terminology by changing "ENSO events" and "ENSO phases" to “ENSO episodes” throughout the text. However, please note that “excess cases” is the outcome variable, and the "probability of excess cases" is mentioned in the results because our exposure and outcome variables are binary, which implies that our estimates are expressed in percentage points in relation to the probability of excess leptospirosis cases.

- Units in ATE: An ATE of 0.072—does this refer to a probability? A proportion of municipalities? Cases per 100,000 population? Clarity here is essential.

R: We appreciate your comment. We have included a new paragraph (lines 302-308) to explain why the units in ATE are expressed as percentage points relative to the probability of excess leptospirosis cases.

- Geographical specificity: While Cali, Barranquilla, and San José del Guaviare are mentioned as high-incidence areas, no maps or spatial analysis are provided. A heatmap or spatial clustering figure would greatly improve interpretability.

R: We appreciate your comment. We have included a new map in Figure 3a to show the municipalities with the highest number of cases.

Questions to Address in Rebuttal:

1. Can you clarify and visualize the actual monthly time series (as claimed) in Figure 2? Or revise the caption to accurately reflect what is shown?

R: We appreciate the comment, and as mentioned above, we have improved the details on the x-axis and highlighted the months with the highest number of cases.

2. What precisely is meant by “excess leptospirosis cases,” and how are they derived for ATE/CATE analysis?

R: As mentioned in a previous answer, lines 136-171 outline the methodology used to estimate and operationalize excess cases, the outcome variable for the estimation of ATE and CATE. To summarize, the first step in identifying excess leptospirosis cases involves calculating the Standardized Incidence Ratio (SIR). This ratio compares the observed number of disease cases within a target population to the expected cases based on a reference population. A SIR value exceeding 1 indicates an occurrence of the disease in the municipality that is higher than expected, while a value below 1 suggests a lower incidence relative to the reference population. To classify excess cases of leptospirosis, we transformed the SIR into a binary variable, assigning a value of 1 when the SIR was above 1 and 0 in all other instances.

3. Please provide methodological transparency on:

- Propensity and outcome model structure

R: As mentioned in a previous answer, we have modified Section 2.5, Machine learning implementation (lines 336-393), to clarify the estimation of propensity score and machine learning model structure.

- Variable selection strategy

R: We have included a new section, 2.3 Variable selection strategy (lines 243-256), to provide methodological transparency regarding the variable selection strategy used in the study. Additionally, we have added new paragraphs (lines 292-301) in Section 2.4, Causal inference analysis, to explain the criteria for selecting a variable in the model adjustment to estimate the effect of ENSO episodes on excess leptospirosis cases.

- How SST was used in CATE estimation

R: In lines 377-389, we describe that we evaluated the CATE only concerning SST12, which refers to the sea surface temperature of the Pacific Ocean along the Colombian coast. This CATE reveals the varying effects of El Niño and La Niña episodes based on the sea surface temperature observed in the Pacific waters off Colombia.

4. Can you reconcile the internal inconsistencies in Table 2? What are the units of the estimates? What do the p-values correspond to?

R: As mentioned in a previous answer, we have modified Section 2.6, Robustness tests in Methods (lines 394-449), and the results (lines 536-563) to clarify the interpretation of Table 2. Specifically, in Table 2, the values shown in "Estimated effect" for each refutation test correspond to the Average Treatment Effect (ATE), while the "New effect" values correspond to the results after introducing the perturbation of the corresponding test, following 50 runs.

5. What is the source and formal method for defining El Niño and La Niña phases? How were transitions handled?

R: As mentioned in a previous answer, we included the definition used by four climate agencies (NOAA, TCC, MOAG, and IDEAM) to identify ENSO episodes. We used the consensus of these four climate agencies as the criterion to define episodes of El Niño, La Niña, and Neutral, because it fulfills the assumption of consistency in causal inference. Note that by using the consensus of these four climate agencies as the criterion to define ENSO episodes, we did not obtain transitions between the episodes.
